# COVID-19-associated impact and post-traumatic stress symptoms 39 days after pandemic in a sample of home-quarantined Chinese college students: the mediating effecting of past stressful events, psychological resilience, and social support

**DOI:** 10.1186/s12888-023-04906-6

**Published:** 2023-05-30

**Authors:** Fanmin Zeng, Wong Chee Meng John, Xueli Sun, Yarong Wang

**Affiliations:** 1 Mental Health Education Centre of Southwest Minzu University, Chengdu, China; 2 Department of Psychological Medicine, National University Hospital & National University of Singapore, Singapore, Singapore; 3 Mental Health Centre of West China Hospital in Sichuan University, Chengdu, China; 4 School of Psychology, Northeast Normal University, 130024 Changchun, China

**Keywords:** COVID-19-associated impact, PTSD, Home-quarantined, Chinese college students

## Abstract

**Background:**

During the COVID-19 outbreak, most Chinese college students were home-quarantined to prevent the spread of the virus. COVID-19-associated impact has been shown to be a risk factor for the development of post-traumatic symptoms disorder (PTSD). However, little is known about the psychological processes that mediate this association. This study investigated the association between COVID-19-associated impact and PTSD and examined whether past stressful events, psychological resilience, and social support have mediating effects on this association.

**Methods:**

The 12,397 valid responses from 31cities in China via an online survey assessed PTSD symptoms, past stressful events, psychological resilience, social support and social-demographic variables. AMOS was used to test the hypotheses of mediating effects.

**Results:**

On the 39th day of the declared COVID-19 epidemic in China, 6.75% of the surveyed sample showed PTSD symptoms. A positive mediating effect of past stressful events was found between COVID-19-associated impact and PTSD, whereas psychological resilience and social support had negative mediating effects. The fit indices for the path model were found to be significant (β = 0.28, *p* < 0.001), COVID-19-associated impact indirectly affects the risk of PTSD through mediating pathways (past stressful events → psychological resilience → social support) on PTSD.

**Conclusions:**

Attention should be paid to the effects of past stressful events of Chinese college students who were home-quarantined during the COVID-19 epidemic, and strategies should also be implemented to improve social support and develop psychological resilience.

**Trial registration:**

The study was approved by the ethics committee of the Southwest Minzu University.

**Supplementary Information:**

The online version contains supplementary material available at 10.1186/s12888-023-04906-6.

## Introduction

The COVID-19 pandemic has been one of the most stressful events in recent times worldwide. Throughout the world, the main method of preventing the spread of COVID-19 has been isolation and social distancing [1]. Many countries began enforcing regional and national containment or lockdown measures starting in January 2020, to prevent the spread of the virus. In China, the implementation of the strict home quarantine measures has kept a large number of people in isolation, particularly Chinese college students who have been unable to return to school due to the pandemic. This situation also raises a number of concerns, including post-traumatic stress disorder (PTSD), which is considered a common adverse outcome in this situation [2, 3]. PTSD is a mental health condition that can occur after experiencing or witnessing at traumatic event, and it can cause symptoms like flashbacks, anxiety, and avoidance behaviors [4]. One such study reported that the prevalence of PTSD in home-quarantined college students was found to be 2.9% [5].

Some studies have suggested that traumatic stress may be due to people's past events [6]. Posttraumatic stress disorder models(pathogenic event models) and the DSM-5 diagnostic criteria [7] also suggest that traumatic stress is a response to past and largely direct exposure to specific life-threatening events, and thus do not readily account for the emerging evidence that COVID-19 is associated with PTSD symptomology. Specifically, Ikizer et al. [8] found that higher levels of perceived stress predicted PTSD symptoms, and that financial stressors, social media use, and time spent at home were associated with higher levels of posttraumatic stress. Therefore, past stressful events may be a driver of PTSD.

Several theories have been proposed to explain how past stressful events may mediate the development of PTSD [9]. One theory is that individuals who have experienced past trauma may have a heightened sensitivity to stress, such that they are more likely to perceive current stressors as threatening and experience greater distress in response to them [10]. Another theory is that past trauma may result in changes in the brain and body that make individuals more reactive to stress and less able to cope effectively with stressors [11]. In the case of COVID-19, past stressful events such as a history of trauma or adverse childhood experiences may increase an individual's vulnerability to developing PTSD symptoms in response to the stressors associated with the pandemic, such as social isolation, financial stress, and fear of illness or death [12]. By understanding the mediate role of past stressful events, mental health professionals can better assess and treat PTSD during the pandemic in individuals who have experienced traumatic events in the past.

The COVID-19 pandemic has had a significant impact on individuals daily lives, including the implementation of social distancing, quarantine, and isolation measures, which may disrupt social support networks [13] and increase the risk of developing psychological symptoms [14]. The Stress and Coping Model explain that individuals rely on coping strategies [15], such as social support, to manage stress during a crisis. However, the pandemic has presented unprecedented challenges that have made traditional coping strategies, including social support, less available, leading to increased stress [16]. Moreover, the pandemic can also impact individuals psychological resilience [17] as it presents significant stressors that challenge an individuals psychological resilience, including fear of infection, financial stress, and uncertainty about future. The Transactional Model of Stress and Coping suggests [18] that an individuals appraisal of stressful situation influences their coping response, and the COVID-19 pandemic may be perceived as a significant stressors that challenges an individuals coping resources, leading to decreased psychological resilience [19].

Psychological resilience can play a mediating role in the relationship between COVID-19 impact and PTSD [20]. Psychological resilience is defined as a multidimensional and dynamic process of successful adaptation to adversity, trauma or significant sources of stress [21]. Ruiz [22] demonstrated that individuals with high psychological resilience can use their coping skills to quickly "bounce back" from distress, while those with low psychological resilience are more susceptible to depressed and feeling overwhelmed. Studies consistently demonstrate that resilience acts as a protective factor against negative psychological outcomes [23], especially during stressful events such as pandemics [24]. For example, research has shown that people with high level of psychological resilience are less prone to depression when faced with stressful events [25] and that it is protective against PTSD [26]. Furthermore, resilience has been found to partially mediate the relationship between mental health and pandemic fatigue, as well as between emotion-oriented coping strategies and general well-being [27, 28]. Overall, psychological resilience is crucial buffer that mitigates the impact of the COVID-19 pandemic on the emotional, psychological, and mental health of individuals [29]. Mental health professionals can use this knowledge to inform the development of effective interventions that promote psychological resilience in individuals affected by the COVID-19 pandemic.

Social support is recognized as a crucial compensatory mechanism that can buffer an individuals psychological responses when confronted with challenging environments such as the COVID-19 pandemic and post-traumatic stress disorder(PTSD) [30], for example, perceived social support was found to mediate the relationship between worry about COVID-19 and psychological health, buffering the negative impact of worry on psychological well-being [31]. It comprises two facets: availability of individuals who can provide potential or actual support in term of personal resources, also known as structural and functional support [32, 33]; and an external coping strategy that enables people to deal with stress by modifying their attitudes towards social support and help-seeking [34]. Prior research indicates that the adequacy of social support is negatively related to the severity of psychological symptoms like depression [35] and positively related to hope [36], resilience [37], post-traumatic growth [38], and has even been identified as a potent protective factor against the development of PTSD following a traumatic event [39]. Consequently, social support could potentially mitigate the stress-psychological symptom nexus during the COVID-19 pandemic.

As mentioned above, previous studies confirmed that psychological resilience and social support are protective factors, and past stressful events is a risk factor for PTSD. However, the COVID-19 pandemic has resulted in a prolonged exposure to stress that is different to the stress caused by disasters in the past. First, there was a rigid coping method such as home quarantine during COVID-19 that could have led to extended exposure to stress. Second, there is uncertainties in the long-term effect of COVID-19 on psychological problems. Therefore, it is important to investigate these mediating factors in the relationship between COVID-19 and the risk of PTSD.

**Fig. 1:**
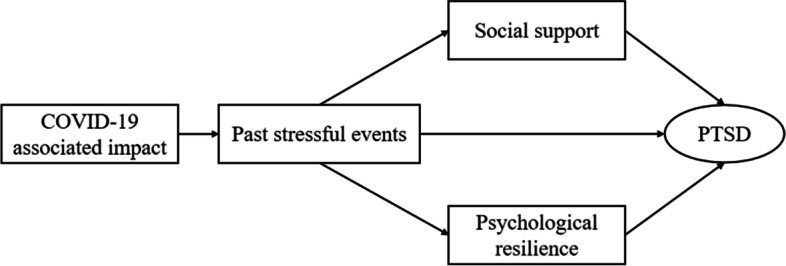
Research hypotheses. The conceptual framework proposed a hypothesis on the relationship between COVID-19-associated impact and PTSD, with consideration for the mediating effects of past stressful events, psychological resilience, and social support

Compared to other social groups, college students are more vulnerable to suffer from the the psychological effects of the COVID-19 pandemic [40]. For college students, social and peer interactions were reduced due to social distancing and stay-at-home requirements, in addition to the uncertainty of future career or academic opportunities due to the lockdown, all of which further exacerbated college students' psychological distress [41, 42]. Therefore, there is an urgent need to pay attention to the psychological problems of college students during the pandemic period. To our knowledge, less studies have investigated the mediating factors in the relationship between COVID-19 and the risk of PTSD in a large sample of the home-quarantined Chinese college students. Such a study would help to fill this gap and provide suggestions for public health management. We hypothesize that the relationship between COVID-19-associated impact and PTSD risk is mediated by past stressful events, psychological resilience, and social support. The hypothesized relationship can be seen in Fig. 1.

## Methods

### Design

The study was conducted in accordance with the principles of the Declaration of Helsinki. It was approved by the ethics committee of the Southwest Minzu University. Anonymous codes were assigned to the self-report questionnaires to ensure confidentiality. Data collection started on 11 March 2020, which was 39 days after the WHO declared the novel coronavirus pneumonia in China as a public health emergency of international concern(PHEIC) [43]. Using the Questionnaire star network platform, we presented the questionnaire online, which was openly accessible to the college students nationwide. The average time to complete the survey was 20 min.

### Participants

The study received a total of 12,397 valid responses by convenient sampling from 31 cities in China by 15 March 2020 through convenient sampling. Inclusion criteria were enrolment as a student, age ≥ 18 years, and home quarantine during the pandemic. Exclusion criteria included those with cognitive and communication impairments that prevented them from completing the survey, and those with acute psychosis or suicidal tendencies. Informed consent was obtained during the online survey.

### Measures

Data were collected from each participant on demographic characteristics: gender, age, year of study (1–4), professional, single-child families, and financial status of students' families.

### PTSD symptoms

The questionnaire used was the Impact of Events Scale-Revised instrument(IES-R) [44]. It is mainly used to measure the level of stress in post-disaster stress disorder and psychological trauma (PTSD). The IES-R consists of 22 items tha are rated on a 5-point Likert scale ranging from 0(not at all) to 4(extremely). The identified trauma symptoms were divided into three categories: (1) flashbacks, intrusive symptoms; (2) avoidance, degenerative symptoms; (3) hypersensitivity, hyperactive symptoms. An IER-S score ≥ 60 indicates severe distress, and a score between 25 to 59 indicates mild distress. In this study, PTSD was diagnosed in college students with total score greater than 60. The COVID-19 Post-traumatic Distress Index inquired about the proximity of the outbreak to the individual's experience, the extent of the epidemic's continuing impact and the severity of the outbreak in the past 1 month. The IES-R has been validated and verified in Chinese with good reliability and validity. Cronbach's alpha is 0.967 for the total scale [45].

### Brief inventory of COVID-19-associated impact

Use of self- created items to assess the COVID-19-associated impact according to the theory of crisis intervention strategies [46]. The inventory of COVID-19-associated impact consists of two parts, which includes 10 items. The first part is assessed on a 5-point Likert scale ranging from 0(none) to 4 (extremely), measure the subjects personal closeness to COVID-19, the extent to which they feel the continuing impact of this pandemic, and the severity of their personal impact by COVID-19. The second part is assessed on a 7-point Likert scale ranging from 0(healthy) to 6(dead), measuring the health status of the subject themselves, family members, relatives, neighbors, friends, teachers, and classmates during the pandemic. The total score is calculated by adding up the scores from both parts of the inventory, and higher scores indicate a more severe of COVID-19- associated Impact. Cronbach's alpha for the total scale is reported as 0.91, indicating good internal consistency.

### Brief inventory of past stressful events

The Essen Trauma Inventory(ETI) [47] is a self-report questionnaire that containing potentially traumatic events(personally experienced or witnessed) and items concerning the objective and subjective threat to life, a total of 14 items including: assault or violence, child abuse, domestic violence, chronic illness, serious psychological problems, legal disputes, substance dependence, gambling addiction, serious car accidents, natural disasters, interpersonal relationship prediction, study pressure, financial distress, emotional out of control, which participants score on a 5-point scale ranging from 4(long-term personal experience) to 0(no experience). The ETI has good psychometric properties, with the Cronbach's alpha of 0.88 (*p* < 0.001) [48].

### Psychological resilience

The original CD-RISC was developed by Connor and Davidson in 2003 [49]. The Chinese version of the CD-RISC was translated and revised from the original CD-RISC by Yu and Zhang [50] in 2007. The Chinese version of the CD-RISC demonstrated good psychometric properties among medical students in previous research [51]. The scale included 25 items and 3 dimensions(tenacity, strength, and optimism), with a Cronbach's a of 0.91 [52]. Each item was scored on a 5-point Likert scale ranging from 0(not true at all) to 4(true nearly all the time), with high scores reflecting high psychological resilience.

### Social support

The 10-item Social Support Rating Scale questionnaire was used to assess social support in our study [53]. This Chinese questionnaire includes three dimensions:(1)objective support, which reflects the support an individual receives in an emergency situation(e.g. “When risk situations are identified, you can receive financial, material, or emotional support from your family members, your close friends, or your colleagues”), (2)subjective support, which reflects an individual's perceived network of friends, neighbors, colleagues, and family members(e.g. “How many close friends do you have?”), and (3)Support use, which refers to the pattern of behaviour an individual uses when seeking social support(e.g. “Do you participate in formal or informal activities?”). Participants are scored on a 4-point scale where the scores ranging from 12 to 66, with higher scores indicating that social support is being provided. The Cronbach' s alpha for social support was 0.83 (*p* < 0.001).

### Statistical analysis

**Fig. 2:**
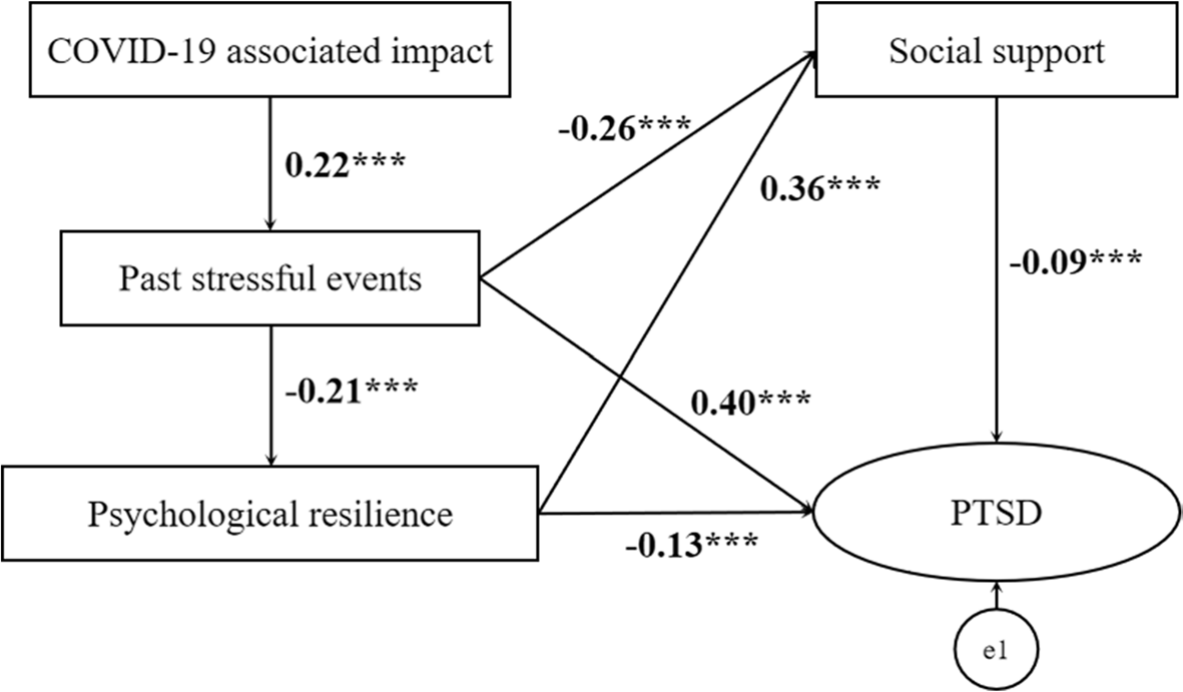
The mediating effects model. Structural equation model-mediating effect of past stressful events, social support and psychological resilience on the association between of COCID-19-associated impact and PTSD symptoms. The number on the lines or arrows is the standardized path coefficient. Arrows indicate the interaction between the variables. The symbol “***” indicates *p* < 0.001

One-way ANOVA or *t-*test was used to examine the associations between the categorical variables. Multiple linear regression analysis was used to identify the predictors of PTSD scale scores, with a *p-*value of less than 0.01 being considered significant. The AMOS24.0 was used to assess the proposed direct or indirect effects of exposure severity on PTSD. Relationships between the variables were assessed using the r-Pearson (r) correlation analysis. According to Cohen, the absolute value of a correlation is equivalent to its effect size, with those below 0.10 being trivial, those between 0.10–0.30 having a small/weak effect, those between 0.30 − 0.50 having a medium effect, and those > 0.50 having large effect [54]. The analysis of the effect of serial mediation (Fig. 2) was performed using the bootstrap method. The sample size for the bootstrap analysis was 5000, and the mediation effect test is significant if it does not contain zero under the 95% confidence interval (CI). The significance level was set at *p* ≤ 0.05.

## Result

### Sample characteristics

This study received a 12,397 valid responses from 31 cities in China by 15 March 2020. Demographic characteristics of the participants are presented in Table 1. Of the total respondents, 4,709 were male (38.04%) and 7,676 were female (61.96%), with an average age of 20.23 ± 1.87 years.

**Table 1:** Descriptive statistics of participants (*N* = 12,397)

Variables	Mean/SD	N/%
Age(years)	20.23/1.87	
Gender		
Male		4709/38.04
Female		7676/61.96
Regional distribution of University		
Northwest Region		4009/32.40
Central China		1968/15.90
Southwest Region		4499/36.30
North China		798/6.40
East China		866/7.00
South China		134/1.10
Northeast Region		32/0.30
Other		73/0.60
Grade		
Freshman		5539/44.80
Sophomore		3339/27.00
Junior		2366/19.10
Senior		594/4.80
Postgraduate		423/3.40
Unknown		118/1.00
Financial status of students families( rmb)		
< 50,000		5899/47.70
50,000–80,000		3216/26.00
80,000–150,000		1919/15.50
150,000–200,000		689/5.60
200,000–300,000		355/2.90
300,000		301/2.40
Single-child families		
Yes		3813/30.80
No		8566/69.20
Professional		
Liberal arts		5282/42.70
Science		5436/43.90
Medicine		655/5.30
Art		926/7.50
Other		80/0.60

### Conceptual variables

Table 2 shows the correlations means, and standard deviations of the five measures in the current samples. The correlation matrix shows that as COVID-19-associated impact (*r* = 0.24; *p* < 0.001), past stressful events (*r* = 0.46; *p* < 0.001) increased, so did symptoms of PTSD increased. In contrast, as social support increased, symptoms of PTSD symptoms decreased (*r* = -0.28; *p* < 0.001). Similarly, as psychological resilience increased (*r *=  − 0.25; *p* < 0.001), symptoms of PTSD decreased.

**Table 2:** Correlation matrix for main variables

	Mean ± SD	COVID-19 associated impact	Past stressful events	Psychological resilience	Social support	PTSD
COVID-19 associated impact	5.71 ± 3.66	1				
Past stressful events	7.61 ± 8.29	0.22**	1			
Psychological resilience	62.95 ± 21.95	-0.03**	-0.21**	1		
Social support	23.84 ± 6.07	-0.06**	-0.33**	0.41**	1	
PTSD	9.98 ± 14.39	0.24**	0.46**	-0.25**	-0.28**	1

^**^p- value < 0.001. Correlation is significant at the 0.001 level (2-tailed)

### Mediation effect of social support and resilience

A series of analyses were conducted to test the first hypothesis on past stressful events, psychological resilience and social support did demonstrate an interaction mediating effect as there was significant associate between COVID-19-associated impact and PTSD (β = 0.28, *p* < 0.001) (Table 3). Confirmatory factor analysis was conducted to analyse the validity and reliability of the proposed mediating instrument. Table 3 shows the fit indices of the mediation model in the mediation model analysis. The χ^2^ value has the property of being sensitive to the sample size [55]. Other fit indices were also confirmed due to the characteristic of χ^2^. The results of the model were χ^2^ = 379.45 (SD = 3.0, *p* = 0.000), CFI = 0.95, GFI = 0.99, RMR = 0.04, RMSEA = 0.08, which were found to meet the criteria for goodness of fit presented in previous studies [56]. Figure 2 illustrates the initial model for the mediating effect of past stressful events, psychological resilience and social support.

**Table 3:** Total direct and indirect effects of Impact of COVID-19 and PTSD

	Effect	Boot SE	Lower 95% CI		Upper95% CI		*p* value	
Past stressful events	0.49	0.00	4.39		5.18		0.001	
Psychological resilience	-0.55	0.00	66.55		67.66		0.001	
Social support	-0.19	0.00	18.62		19.44		0.001	
PTSD	0.70	0.01	13.87		16.38		0.001	
Indirection e1	0.28	0.06	149.74		164.88		0.001	
Default model	χ^2^	CFI	GFI	RMR	NFI	IFI	TLI	RMSEA
	379.45	0.95	0.99	0.04	0.95	0.95	0.91	0.08

*CFI* Comparative fit index, *GFI* Goodness of fit index, *RMR* Root mean square residual, *NFI* Norm fit index, *IFI* Incremental fit index, *TLI* Tucker-Lewis index, *RMSEA* Root mean square error of approximation

## Discussion

Our study focused on the mediating effects in the association between COVID-19-associated impact and PTSD in home-quarantined Chinese college students. A positive mediating effect of past stressful events was found between COVID-19-associated Impact and PTSD, whereas psychological resilience and social support had negative mediating effects. Path model fit indices for the path model were found to be significant (β = 0.28, *p* < 0.001), indicating that COVID-19-associated impact indirectly affects the risk of PTSD through mediating pathways (past stressful events → psychological resilience → social support) on PTSD. Our results are consistent with previous studies [57, 58], which found that participants experienced traumatic stress symptoms as a result of COVID-19, and this was associated with direct exposure to past events that threatened death, injury or other violation. Although the stressful event is not a diagnostic criterion for PTSD in DMS-defined traumatic stress [59, 60], it can still result in levels of traumatic stress equal to or greater than of PTSD, which may explain why exposure to events such as government lockdown was associated with traumatic stress symptoms. Therefore, we should pay more attention to the impact of past stressful events on the risk of PTSD symptoms during a pandemic.

Other than the mediating effect of past stressful events, a large body of existing research has also revealed the mediating effects of social support, and psychological resilience on the association between traumatic experiences and psychological morbidity. The findings were consistent with previous studies. Accumulating evidence suggests that social support is an effective emotional regulator under conditions of traumatic stress and contributes to a reduced risk of or protection against PTSD [61, 62]. On the contrary, lower levels of social support are strongly associated with higher risk of PTSD [48]. There are two possible explanations for this: first, social support is an important resource for people to overcome the distress, allowing them to cope with the traumatic experience in a positive ways [63]; second, seeking social support is a way of coping with adversity and is considered a problem-focused coping strategy that has been shown to be effective in reducing stress. When stress and anxiety are elevated, adequate social support may help college students to maintain in healthy emotional states. Psychological resilience is defined as a personal resource for coping with or overcoming a variety of adversities and perceived stress [64]. Karatzias [65] reported that psychological resilience mediates the relationship between traumatic experiences and post-traumatic adjustment, and Duncan [66, 67] found that during stressful events, individuals are more likely to experienced negative mental and psychological outcomes if they are not equipped with sufficient levels of resilience and coping skills. In addition, several studies have found a positive association between social support and psychological resilience [5, 68], with social support being associated with greater psychological resilience and lower PTSD symptomology. This suggests that increasing social support may be a strategy for enhancing psychological resilience.

In our study, the mean (SD) PTSD (IES-R) score of the sample was 31.98 ± 14.39. Almost 53.87% of the respondents experienced posttraumatic distress ( 47.12% of the respondents scores were between 25 and 59, and 6.75% of the respondents' scores were ≥ 60). Compared to a previous study using a sample of 2485 Chinese college students quarantined after the outbreak of the COVID-19 epidemic, our PTSD rates were found to be much higher (6.75% vs 2.70%) [69]. A possible explanation could be the different study designs, the use of participants from different counties, and the different assessment methods. Multinomial logistic regression analyses showed that one's post-traumatic distress scores were associated with their gender, grade, professional and region (*R*^2^ = 0.06, *F* = 80.54, *p* < 0.001). Female respondents reported significantly higher levels of post-traumatic distress than their male counterparts(mean(SD) = 13.53(4.70) vs 13.05(5.11), *p* < 0.001). This is consistent with the findings of previous research concluding that women are much more vulnerable to stress and more likely to develop post-traumatic stress disorder [70, 71]. Of all the students, the medical students had the highest scores on the IES-R mean (SD) = 32.23(13.50). A positive explanation could be that medical students have greater medical knowledge of novel coronavirus pneumonia, which means more stress on the epidemic during exposure. This is in line with previous research [70]. It is noteworthy that students from single-child families also experienced the highest level of post-traumatic distress mean (SD) = 32.11(14.38, t = 3.32, *p* < 0.05,). This may be due to a lack of interaction with family members of a similar age group during home-quarantine, but needs to be confirmed with further research. Meanwhile, the financial status of students' families is also an influencing factor on the IESR scores(the lower the annual income, the greater the pressure on families)(mean (SD) = 32.28(14.89), *F* = 2.71, *p* < 0.05). The financial status of the students' families can be partly attributed to the effective prevention and control measures taken by an important social support, as this social support is considered to be one of the most important protective factors in reducing PTSD symptoms in individuals after traumatic events [71].

The study has some limitations that should be noted. First and foremost, the current analysis used cross-sectional data, so no causal claims can be made. In addition, we did not examine the long-term effect of COVID-19-associated impact on the risk of PTSD. Nevertheless, we used a large sample size covering more than 30 cities in China; this convenient sampling method may have introduced a bias in the diversity of the sample and resulted in underrepresentation of respondents. Finally, this study examined the mediating effects in the association between COVID-19-associated impact and PTSD in home-quarantined Chinese college students and did not compare the situation when the participants returned to campus or the effects of the ongoing, prolonged nature of the pandemic.

## Conclusions

Our findings highlight the impact of the past stressful events on PTSD, suggesting that assessment of stressful events in students during the pandemic is necessary to reduce the risk of PTSD. Recommendations for further interventions include: (1) more attention needs to be paid to those who have experienced past stressful or negative events in the past, medical students, female students, students from single-child families and those with lower financial status, and by providing a sense of greater emotional security, thereby reducing their stress levels and enabling them to adapt effectively during the pandemic; (2) provide more social support needs to be provided to college students suffering from psychological distress by implementing theory-tested interventions or online programme, such as online workshops and on-demand counselling. This will increase the opportunities for students to express their feelings and concerns and openly discuss their experiences and challenges with their families; (3) establish a comprehensive psychological curriculum system in universities or colleges in China should be built to develop psychological resilience and promote the level of mental health among college students.

## Supplementary Information

**Additional file 1. **

## Data Availability

Search the supplementary information files or send an email to zengfanmin929@163.com.

## Abbreviations

PTSD: Post Traumatic Symptoms Disorder
ETI: The Essen Trauma Inventory
CFI: Comparative Fit Index
GFI: Goodness of Fit Index
RMR: Root Mean Square Residual
NFI: Norm Fit Index
IFI: Incremental Fit Index
TLI: Tucker-Lewis Index
RMSEA: Root Mean Square Error of Approximation
